# Mild Drought Promotes Biomass Accumulation and Increases Diosgenin Content in Rhizomes of *Dioscorea nipponica*

**DOI:** 10.3390/plants14192998

**Published:** 2025-09-28

**Authors:** Ran Wang, Zhigang Xue, Zixing Li, Huan Cao, Jiayu Wang, Runze He, Haoyuan Gao, Runmei Gao

**Affiliations:** College of Forestry, Shanxi Agriculture University, Jinzhong 030801, China; 18536494038@163.com (R.W.); 15596030223@163.com (Z.X.); lizixing0927@163.com (Z.L.); ttw610610@163.com (H.C.); 13233557416@139.com (J.W.); 19722729712@139.com (R.H.); 19722726131@163.com (H.G.)

**Keywords:** *Dioscorea nipponica*, diosgenin, environmental factors, drought stress, plant growth

## Abstract

*Dioscorea nipponica* is an important medicinal and edible plant in northern China, and its extract dioscin is an important raw material for the modern pharmaceutical industry. To investigate the key environmental factors influencing diosgenin accumulation in the rhizomes of *D. nipponica* and their action mechanism, we collected wild *D. nipponica* plants from 60 plots on Zhongtiao Mountain and analyzed the effects of environmental conditions on both plant growth and diosgenin synthesis. Additionally, physiological parameters of *D. nipponica* were investigated under different intervals of watering treatments: control (CK, 3 days), mild drought (MID, 5 days), moderate drought (MD, 8 days) and severe drought (SD, 10 days). The results showed that the water content of rhizome was the main factor affecting the diosgenin content, and soil nutrients, especially nitrogen, played an important role in the growth of *D. nipponica*. The results of a drought stress gradient test showed that diosgenin increased significantly under mild drought compared to the control, reaching 103.19 ± 2.63%. SD inhibited the growth of plants, and the indexes decreased by 10.08 ± 0.03–34.94 ± 5.60% compared with MID but increased the proliferation rate of rhizomes (83.33%), which is the reproductive strategy of *D. nipponica* when faced with drought stress. It provides a scientific basis for imitation of wild cultivation of *D. nipponica*.

## 1. Introduction

*Dioscorea nipponica* is an important medicinal and edible plant in northern China, and its extract diosgenin is an important raw material for the modern pharmaceutical industry. More than 60% of steroid hormones are made from this raw material, which is called ‘medicinal gold’ [1]. In recent years, studies have shown that diosgenin has a wide range of pharmacological action and important biological activities, such as anti-tumor, prevention of cardiovascular and cerebrovascular diseases, treatment of rheumatoid arthritis and hypoglycemic and immune regulation [2,3,4,5]. With the development of the pharmaceutical industry, the market prospect of diosgenin is increasingly broad [6]. The supply of *D. nipponica*, as one of the main raw materials for the production of diosgenin in China [7,8], has long been dependent on wild medicinal materials. With the increasing demand for diosgenin, the excavation cycle and resource recovery period of *D. nipponica* have been forced to shorten [9]. However, the understory imitation wild cultivation technology of *D. nipponica* is not mature. What factors mainly affect the synthesis of diosgenin in the rhizome of *D. nipponica* and how environmental factors affect the growth and development of *D. nipponica* have not been elucidated. Therefore, it has become the first choice to protect wild resources and improve the quality and value of medicinal materials by studying the influence of the environment on *D. nipponica* growth and diosgenin content, improving the wild-like cultivation technology in forests and cultivating elite plants.

The yield of specialized metabolites in medicinal plants is significantly influenced by their natural environments [10]. Previous studies have shown that latitude, annual average temperature and annual precipitation will significantly affect the accumulation of diosgenin in Dioscorea zingiberensis [11], and the gene *DzCYP72A12-4* related to diosgenin synthesis can also improve plant drought adaptability [12]. Although the rhizome is the main medicinal part as the accumulation site of diosgenin, the synthesis of diosgenin is completed in the leaves [13], and there is also an epimer of diosgenin in chloroplasts of *D. zingiberensis* [14]. Light and photosynthesis may be closely related to the synthesis of diosgenin. In the study of fenugreek, diosgenin was pointed out to be affected by high-temperature, low-temperature and salt stress, together with various antioxidant pathways as a defense strategy for plants [15,16,17].

Drought is one of the main limiting factors that strongly affect the growth, development and secondary metabolic processes of rhizome medicinal plants [18,19]. Many studies reported that drought can stimulate the increase in secondary metabolites. The growth and development of *Paris polyphylla* are hindered under severe drought (WC40), but the content of steroidal saponins in *Paris polyphylla* is greatly increased [20]. *Panax ginseng* also has a similar performance, drought stress inhibits *Panax ginseng* root biomass [21], but it promotes the synthesis of ginsenosides to regulate the synthesis and metabolism of plant photosynthetic products and adapt to an arid environment [22]. The yield and saponin content of *Panax notoginseng* were more synchronized [23], and mild drought treatment could not only reduce the occurrence of root rot of *Panax notoginseng* but also improve the water use efficiency, saponin content and economic benefits of *Panax notoginseng* [24].

In this study, 60 groups of *D. nipponica* on Zhongtiao Mountain of Shanxi Province were investigated, and the growth factors and diosgenin content of *D. nipponica* in groups were determined. The effects of 21 indicators, including abiotic factors (altitude, slope direction, slope, soil nutrients), biological factors (community type, biodiversity, canopy density, shrub coverage, herb coverage) and plant growth factors (plant number, plant height, ground diameter, rhizome dry weight, rhizome water content), on diosgenin content were analyzed.

The purpose of this study was to (1) determine the main environmental factors affecting wild *D. nipponica* by using the effective causal analysis of PLS-PM; (2) after screening out the key factor of water, the differences in growth, physiology and diosgenin content of *D. nipponica* seedlings under different drought stresses were observed, and which condition was most suitable for diosgenin synthesis was determined. The results of this study will provide a good basis for improving the adaptability of *D. nipponica* to the environment and selecting the best way to carry out large-scale understory planting.

## 2. Results

### 2.1. Effects of Environment on Growth and Diosgenin Content of D. nipponica

#### 2.1.1. Effects of Environmental Factors on Growth

**Figure 1:**
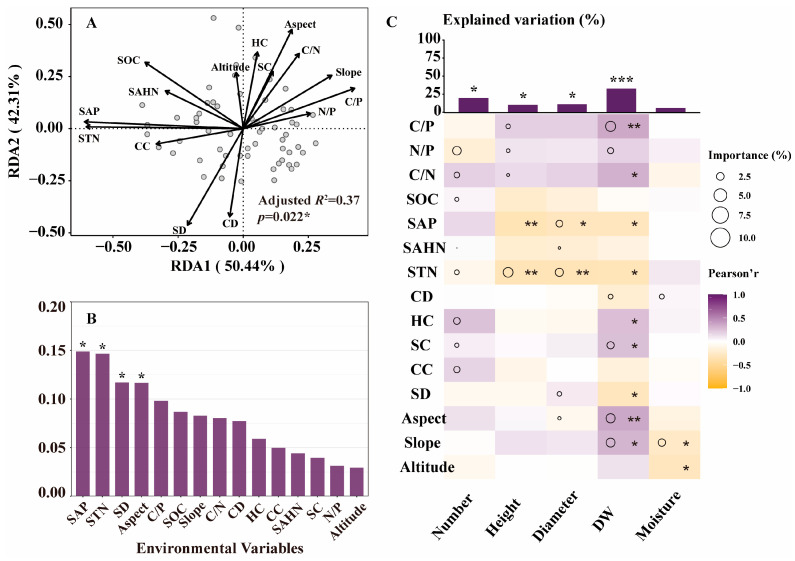
The effects of environmental factors on growth of *D. nipponica* (*n* = 60). (**A**) shows the relationship between growth index and environmental factors. (**B**) shows the contribution of environmental factors based on RDA models. (**C**) shows the influence of environmental factors on growth index respectively. Asterisks denote the level of significance (* *p* < 0.05, ** *p* < 0.01, *** *p* < 0.001). Number, community stem count; Height, plant height; Diameter, ground diameter; DW, rhizome dry weight; Moisture, water content of rhizome; C/P, carbon to phosphorus ratio; N/P, nitrogen to phosphorus ratio; C/N, carbon to nitrogen ratio; SOC, soil organic carbon; SAP, soil available phosphorus; SAHN, soil ammonium hydroxide nitrogen; STN, soil total nitrogen; CD, community diversity; HC, herbaceous cover; SC, shrub cover; CC, canopy closure; SD, stand density.

Redundancy analysis (RDA) showed that environmental factors were closely related to the growth index of *D. nipponica* (*R*^2^ = 0.37, *p* < 0.05). The first two RDA axes explained 50.44% and 42.31% of the total variation in the growth index of *D. nipponica*, respectively, (Figure 1A). SAP (*p* < 0.05), STN (*p* < 0.05), SD (*p* < 0.05) and Aspect (*p* < 0.05) were significantly correlated with changes in growth indicators (Figure 1B). The multiple regression model showed that environmental factors were significantly correlated with Number (*p* < 0.05), Height (*p* < 0.05), Diameter (*p* < 0.05) and DW (*p* < 0.001) but could not explain the change of Moisture. Combining the correlation analysis of response variables and environmental factors, SAP mainly affected the change of Diameter and was significantly negatively correlated with Height (*p* < 0.01), Diameter (*p* < 0.05) and DW (*p* < 0.01). STN mainly affected the changes of Height and Diameter and was significantly negatively correlated with Height (*p* < 0.01), Diameter (*p* < 0.01) and DW (*p* < 0.01). SD mainly affected the change of Diameter and had a significant negative correlation with DW (*p* < 0.05). Aspect mainly affected the changes of Diameter and DW and was significantly negatively correlated with DW (*p* < 0.01).

#### 2.1.2. Effects of Environmental Factors on Diosgenin

**Figure 2:**
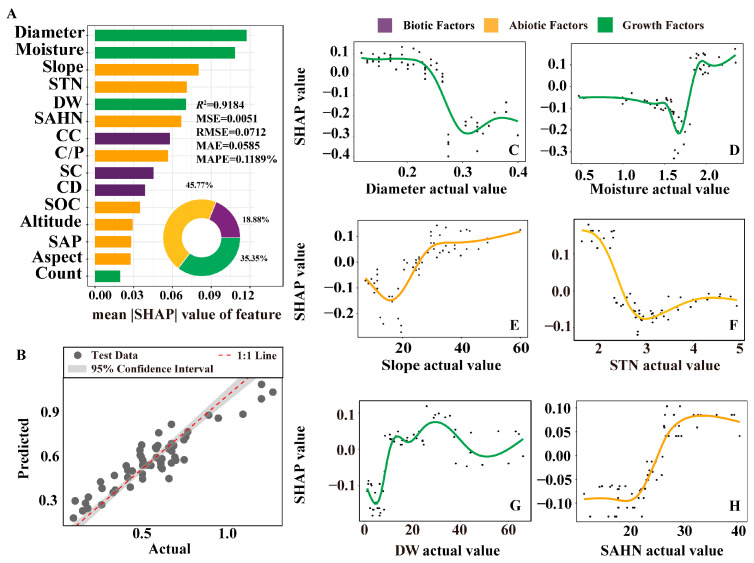
Effects of environmental and growth physiological indexes on diosgenin content (n = 60). (**A**) is factor importance analysis based on the XGBoost-SHAP model and shows the contribution of biological (purple), non-biological (yellow) and plant growth (green) indicators to the content of diosgenin. (**B**) is a comparison of the actual and predicted diosgenin content based on the XGBoost model. (**C**–**H**) are smoothed splines fitted based on the distribution of these SHAP values. Diameter, ground diameter; Moisture, water content of rhizome; STN, soil total nitrogen; DW, rhizome dry weight; SAHN, soil ammonium hydroxide nitrogen; CC, canopy closure; C/P, carbon to phosphorus ratio; SC, shrub cover; CD, community diversity; SOC, soil organic carbon; SAP, soil available phosphorus; Count, community stem count.

The XGBoost-SHAP model was fitted with community information, geographic information, soil nutrient information and plant growth information as input variables. The prediction of diosgenin by the model showed high goodness of fit in all indicators, the *R*^2^ reached 0.9184 (Figure 2A) and the comparison between the actual value and the predicted value of diosgenin also showed that the model could accurately predict the trend of the data (Figure 2B). The abiotic factors in the model had a significant impact (45.77%), and environmental factors such as Slope, STN and SAHN were at the top of the ranking and had a greater impact. The influence of growth status was the second (35.35%), and the variance interpretation rate of the two indexes of ground diameter and rhizome water content was the highest, which was the main factor affecting the content of diosgenin. The effect of biological factors on the content of diosgenin was the smallest (18.88%) (Figure 2A).

There are non-linear relationships affecting the diosgenin content of *D. nipponica* in terms of biotic and abiotic factors such as diameter, moisture, vegetation coverage (CC and SC), slope and soil nutrients (STN, SAHN and C/P). The non-linear fitting of the top six factors in the XGBoost-SHAP model is performed to show the relationship between the SHAP value and the actual value (Figure 2C–H). Among the abiotic factors, the Slope changed to a positive effect at 23°, increased rapidly at 20–26° and showed a continuous positive effect after exceeding 26°, but the degree of increase began to slow down. The effect of soil nutrients on diosgenin content was mainly reflected in N element. STN had a positive effect on diosgenin at low concentration and turned into a negative effect after 2.5 g·kg^−1^. The trend of SAHN was opposite to that of STN, showing low concentration inhibition and high concentration promotion. In the growth factors, the growth was promoted when the diameter was small and inhibited when the diameter was large, with a turning point of 0.24 cm. Low moisture inhibits diosgenin accumulation, with the strongest inhibitory effect observed at 1.6% moisture, then it quickly changed to a promotion effect and tended to be stable at 1.9%. The dry weight showed a single peak curve as a whole, and too high or too low DW would inhibit the synthesis of diosgenin.

### 2.2. Analysis on the Difference of Indexes of D. nipponica Under Different Drought Stresses

#### 2.2.1. Effects of Drought Stress on Growth

Under different drought stresses, seedling survival rate (SR) and rhizome proliferation rate (PR) of *D. nipponica* seedlings were significantly affected, while Height and Diameter remained at the same level (Table 1). With the increase in drought stress, the SR, Height and Diameter of *D. nipponica* showed a trend of increasing first and then decreasing, with the highest at MID and the lowest at SD. Compared with CK, the above indexes increased by 13.5 ± 0.7%, 16.9 ± 0.8% and 27.5 ± 5.6%, respectively, at MID. The PR variation trend was decreasing first and then increasing, with the lowest value (0.18 ± 0.04) at MID and the highest value (0.33 ± 0.04) at SD. These results suggest that appropriate drought stress can promote the growth and survival of *D. nipponica* but is not conducive to its rhizome proliferation.

#### 2.2.2. Effects of Drought Stress on Physiological Parameters

The leaves of *D. nipponica* were used as test materials, and the changes of oxidative stress indexes (SOD, CAT, MDA), osmotic adjustment substances (Pro, SS, SP) and photosynthetic characteristics (Chl, Pn, Tr, Gs, Ci, WUE) were studied (Table 2). As one of the products of membrane lipid peroxidation, the change of MDA content can measure the damage degree of plant cell membranes under adversity; antioxidant enzymes are the key to scavenging ROS, which can protect plant cells from oxidative damage [25]; osmotic regulators are a class of solutes that are actively accumulated by plants under drought stress. They can maintain water balance by regulating cell osmotic potential and enhance plant drought resistance [26]; photosynthesis is the basis of plant growth and yield formation. Drought stress disrupts plant carbon metabolism and is an important environmental factor that inhibits photosynthesis [27]. SOD and SS increased first and then decreased with the increase in drought degree. Compared with CK, MID increased them by 107.09 ± 10.21% and 53.51 ± 4.47%, respectively, and in SD, they increased by 52.74% and 24.14%, respectively. The trend of other physiological indexes was significant. CAT, MDA, Pro and Ci increased significantly with the increase in drought stress. Compared with CK, SD increased by 48.99 ± 0.63%, 182.01 ± 1.27%, 122.28 ± 8.49% and 196.89 ± 8.69%, respectively. SP, Pn, Tr, Gs and WUE decreased significantly with the increase in drought stress. Compared with CK, SD decreased by 17.77 ± 4.00%, 84.17 ± 4.84%, 64.81 ± 8.97%, 73.18 ± 9.33% and 33.63 ± 0.64%, respectively.

#### 2.2.3. Effects of Drought Stress on Rhizomes

Under different drought stresses, DW, Moisture and Dio of rhizomes were significantly affected (Table 3). With the aggravation of drought stress, DW, Moisture and Dio increased first and then decreased. Compared with CK, DW increased under three drought treatments and was the highest at MID, which was 0.79 g. The trend of Moisture and Dio was MID > CK > MD > SD. And surprisingly, Dio of MID was as high as 6.5 mg·g^−1^, which was 103.19 ± 3.23% higher than that of CK. The effect of severe drought on Dio was also greater, and SD decreased by 65.67 ± 2.05% compared with CK.

## 3. Discussion

### 3.1. The Key Factors Affecting the Growth and Diosgenin Content of Wild D. nipponica

For the growth of *D. nipponica*, soil nutrients and light are the two main influencing factors. SAP, STN, C/P and other soil nutrient factors have the greatest impact. SAP and STN were significantly negatively correlated with Height, Diameter and DW of *D. nipponica* (Figure 1). This indicates that high concentration of SAP will inhibit the growth of *D. nipponica*, which may be due to the key role of *D. nipponica* endophytes in the process of phosphorus absorption [28,29]. Excessive SAP will inhibit the activity of endophytes [30], which in turn inhibits plant growth. The study also found that SD, Aspect and other light-related environmental factors play a very important role. SD has a negative effect, and Aspect has a positive effect, indicating that the growth of *D. nipponica* needs sufficient light. The positive correlation between SC and DW indicates that, as a shade-loving plant, a certain amount of cover is necessary. Plants can avoid high radiation damage under moderate shading, but excessive shading will limit their stem and leaf development and photosynthesis due to insufficient light energy [31].

**Figure 3:**
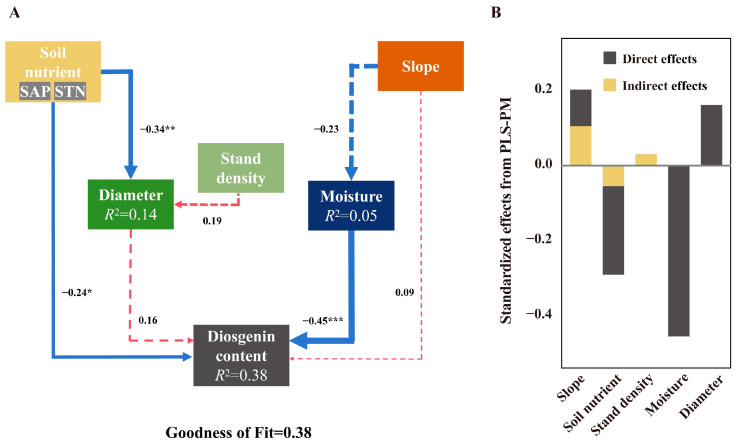
The relationship between diosgenin content in rhizome of wild *D. nipponica* and environmental factors and growth status (*n* = 60). (**A**) is the PLS-PM plot of the effects of Soil nutrient, Slope, SD, Diameter and Moisture on Diosgenin content. Red solid arrows indicate significant positive effects, blue solid arrows indicate significant negative effects and dashed arrows indicate non-significant effects. The model was evaluated using the goodness-of-fit (GoF) statistic, which is a measure of overall predictive performance. The GoF statistics above the cutoff values of 0.1, 0.25 and 0.36 were classified as weak, moderate and strong, respectively. (**B**) is the standardized direct, indirect and total effects of each predictor on diosgenin content in the PLS-PM used. Asterisks denote the level of significance (* *p* < 0.05, ** *p* < 0.01, *** *p* < 0.001).

Combined with the results of the XGBoost-SHAP model and RDA model, the fitted PLS-PM model (GoF = 0.38) showed that water conditions (β = −0.45, *p* < 0.001) would directly affect the accumulation of diosgenin, while soil nutrient status (β = −0.24, *p* < 0.05) directly affected the accumulation of diosgenin on the one hand and indirectly affected the content of diosgenin in tubers of *D. nipponica* by changing plant growth status (β = 0.16) on the other hand (Figure 3). The results showed that Moisture had a significant inhibitory effect on the content of diosgenin, while Slope reduced Moisture on the one hand and promoted the synthesis of diosgenin on the other hand. The increase in slope gradient will increase the surface runoff and lead to a corresponding decrease in soil water holding capacity [32], which indicates that water conditions, especially drought stress, may be the main factor inducing diosgenin synthesis. In addition to water, soil nutrient conditions also play a crucial role in diosgenin accumulation. In terms of plant growth and diosgenin synthesis, they showed a trend of low concentration promoting high concentration inhibition, but SAHN had no significant inhibition on growth and even promoted the synthesis of diosgenin, which further indicated that the ability of *D. nipponica* to directly absorb soil nutrients was weak, and it could only be absorbed after the transformation of soil microorganisms or endophytic bacteria. The growth status is also related to diosgenin content. Diameter less than 0.24 cm will promote the synthesis of diosgenin, but a diameter greater than 0.24 cm will inhibit the synthesis of diosgenin. This is very similar to Panax notoginseng. The content of ginsenosides in both good and bad growth is not as high as for medium growth, and primary metabolism (starch and sucrose metabolism) plays a very important role in this process [33].

Although the above models, especially the XGBoost-SHAP model, show promising predictive performance, our research has some limitations that need to be acknowledged. First of all, the samples in this paper are only collected from the Zhongtiao forest area in Shanxi Province, which has regional specificity. In addition, although we used cross-validation to reduce the overfitting risk of the model, the limited sample size (n = 60) may not be sufficient to capture the complex non-linear relationships between diosgenin content and many environmental factors. Therefore, when the model is applied to other regions, there may be a risk of a significant decline in its predictive performance, and more extensive verification is needed to regulate the universal ecophysiological laws of diosgenin accumulation.

### 3.2. Effect of Drought Stress on D. nipponica

#### 3.2.1. Effects of Drought Stress on Growth

In this study, drought stress significantly affected the key physiological parameters of *D. nipponica*. Faced with different degrees of drought stress, the physiological response of *D. nipponica* seedlings can be divided into two stages. The first stage is from no stress to mild stress. Under mild drought, the growth index of *D. nipponica* seedlings except the proliferation rate increased to varying degrees. The second stage is from mild stress to severe stress. As the degree of stress deepens, the plants show obvious poor growth, and the rhizome increment rate increases, which is opposite to the trend of growth index.

In the first stage, the key physiological parameters of *D. nipponica* showed a variety of behaviors to adapt to drought and reduce the negative effects of drought. First, there is an increase the synthesis of antioxidant enzymes (SOD, CAT) to remove ROS caused by the destruction of the electron transport chain in plants [25] and reduce the damage of oxidative stress to cells. SOD can decompose O_2_^−^ into O_2_ and H_2_O_2_, which eliminates the formation of •OH through the Haber–Weiss reaction [34]. CAT is usually used to catalyze the rapid decomposition of H_2_O and O_2_ [35]. Drought stress activated the activities of SOD and CAT, and the response of SOD to mild stress was stronger, with an increase of about three times that of CAT. At this time, although MDA increased to a certain extent, it was not significant. The increase in antioxidant enzymes effectively controlled the degree of oxidative damage to membrane lipids and protected the integrity of cell membranes [36]. Secondly, by increasing Pro, SS, SP and other osmotic adjustment substances, the osmotic potential of the cells was reduced, and the cell turgor was maintained, so that the plants could still absorb water from the soil under drought conditions. At this time, the water content of the rhizome of *D. nipponica* increased by 5.98%. Finally, WUE was improved by reducing Tr and Gs. In addition, although Chl and Ci increased significantly under drought stress, Pn continued to decline, which was contrary to the phenomenon that plants grew well. In view of the fact that the photosynthetic indexes of plants in this experiment were measured during the day, it may be due to the fact that *D. nipponica* also has a defensive behavior similar to the facultative CAM pathway in the face of drought stress. Facultative CAM refers to the ability of some plants to switch from the C_3_ or C_4_ photosynthetic pathway to the CAM pathway under specific environmental conditions, generally drought [37]. These plants open stomata at night to absorb CO_2_ and convert it into malic acid, store CO_2_ in vacuoles and release CO_2_ during the day to complete photosynthesis, balancing growth needs and drought tolerance through such dynamic regulation [38], but this remains speculative and requires further experimental validation. The decrease in PR may be due to the rapid synthesis of osmotic protective substances and the activation of the ABA signaling pathway during drought stress to maintain cell survival [39], but this process will inhibit the expression of auxin synthesis genes in plants, resulting in poor main root growth [40]. The above measures have significantly improved the survival rate, rhizome dry weight and water content of *D. nipponica* seedlings in the face of drought stress and reduced the impact of drought stress on plant survival and growth.

In the second stage, *D. nipponica* will also reduce the effects of drought stress on plant growth and metabolism through the above methods. However, on the one hand, these measures have been unable to effectively control drought damage. MDA increased significantly, WUE and Moisture decreased uncontrollably, and compared with MID, the growth index of *D. nipponica* decreased by 10.08 ± 0.03–34.94 ± 5.60% during SD. On the other hand, the strategies of plant defense and growth have also changed, and the survival of plants has been challenged. The gradual deterioration of the living environment also makes *D. nipponica* unable to maintain the physiological costs of the immune system [41]. SOD, SS, SP and Chl decreased successively after the peak of content, and PR began to rise. This may be because, when faced with severe stress, rhizome plants will accelerate tuber formation or increase the distribution of asexual propagules, and their rhizomes are buried deep underground and are less affected by drought. The stored starch and water can support the germination of new divisions after rainfall [42]. It is a survival strategy for plants to face severe stress by sacrificing individual survival for population continuation.

In addition, our study also observed that, although the growth indexes decreased significantly at SD, DW still showed an increase compared with CK, and DW/Diameter increased from 0.96 ± 0.13 to 1.26 ± 0.25. This shows that *D. nipponica* will allocate aboveground and underground biomass in the face of severe drought [43]. This phenomenon is consistent with the upward trend of PR, which further indicates that the change of biomass allocation of *D. nipponica* is a survival strategy to cope with SD. It tends to prioritize the allocation of limited resources to the underground part, that is, underground clonal reproductive organs. This finding is consistent with the stress response strategies of many clonal plants. For example, *Phragmites communis* maintains population stability by increasing underground biomass and ramet number in wind erosion environments [44]; the hyperactivation of jasmonic acid signaling in *Panax ginseng* inhibits IAA-mediated growth pathways and shifts carbon flux to the synthesis of defensive secondary metabolites (such as ginsenosides) [45]. These results collectively indicate that, when resources are limited, perennial herbs generally maintain individual inventory and population resilience by increasing the investment in storage organs or clonal propagules. Therefore, it may partly explain why poor seedling growth occurred during severe drought but the rhizome value-added rate still increased.

#### 3.2.2. Effects of Drought Stress on Diosgenin

**Figure 4:**
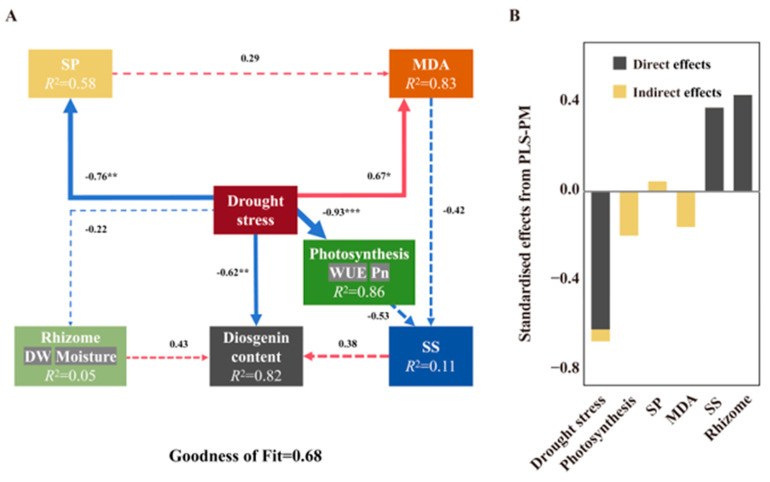
Relationships between physiological parameters and content of diosgenin in rhizomes of *D. nipponica* (*n* = 12). (**A**) is the PLS-PM plot of the effects of drought stress, SP, MDA, photosynthesis, SS and rhizome on diosgenin content. Red solid arrows indicate significant positive effects, blue solid arrows indicate significant negative effects and dashed arrows indicate non-significant effects. The model was evaluated using the goodness-of-fit (GoF) statistic, which is a measure of overall predictive performance. The GoF statistics above the cutoff values of 0.1, 0.25 and 0.36 were classified as weak, moderate and strong, respectively. (**B**) is the standardized direct, indirect and total effects of each predictor on diosgenin content in the PLS-PM used. Asterisks denote the level of significance (* *p* < 0.05, ** *p* < 0.01, *** *p* < 0.001).

PLS-PM provided additional information, indicating that drought stress was mainly a direct effect (β = −0.62, *p* < 0.01), partly through indirect effects of plant growth (β = 0.43) and sugar metabolite SS (β = 0.38) (Figure 4). This may be because saponins play an important role in regulating plant stress resistance. In the study of *Ophiopogon japonicum*, Cd stress leads to the accumulation of total saponins. The antioxidant capacity of saponins can inhibit Cd-induced reactive oxygen species (ROS) accumulation and promote SOD activity [46]. In vitro experiments of *Agave salmiana* also proved that drought stress increased the content of saponins and antioxidant activity [47]. Other studies have shown that dioscin also has the ability to reduce oxidative stress damage [48]. Therefore, it is speculated that diosgenin can also reduce the antioxidant damage of plants through similar mechanisms in the face of drought stress. In addition, the application of mild drought stress is an effective strategy to enhance the accumulation of dioscin in the rhizomes of *D. nipponica*. However, from the perspective of practical cultivation, it may be difficult to apply irrigation management in the field and control mild drought conditions. The use of mild salt stress (such as KCl) to induce dioscin accumulation may be an alternative and easier to manage. Drought and salt stress have similar physiological mechanisms, and both activate the antioxidant system through osmotic stress [49,50]. Compared with drought stress, K^+^ can alleviate the negative effects of osmotic stress on plant growth to a certain extent [51]. Therefore, it has more operability and application advantages in field management and can be further explored in the related research of Dioscorea nipponica in the future.

Although mild stress will greatly increase the content of diosgenin, the inhibitory effect of severe stress makes drought stress negatively correlated with the content of diosgenin in rhizomes, which may be related to the structural adjustment of carbon in *D. nipponica*. Previous studies have shown that glucose metabolism is closely related to saponin biosynthesis, including substrate supply, energy supply and regulation of specific gene expression [52]. The synthesis of diosgenin starts from acetyl-CoA [53]. Acetyl-CoA is an important intermediate product of saccharometabolism, and saccharometabolism is the upstream metabolism of diosgenin synthesis. It was found in the study of the saponin synthesis pathway in *Platycodon grandiflorum* that MeJA could induce the expression of genes related to glycolysis, starch and sucrose metabolism, thereby promoting the biosynthesis of triterpenoid saponins [54]. Under severe stress, the sugar metabolism of plants is blocked, and the content of SS decreases. The lack of a carbon source and energy supply also inhibits the synthesis of secondary metabolites and reduces the content of diosgenin.

## 4. Materials and Methods

### 4.1. Study Areas and Sample Collection

**Figure 5:**
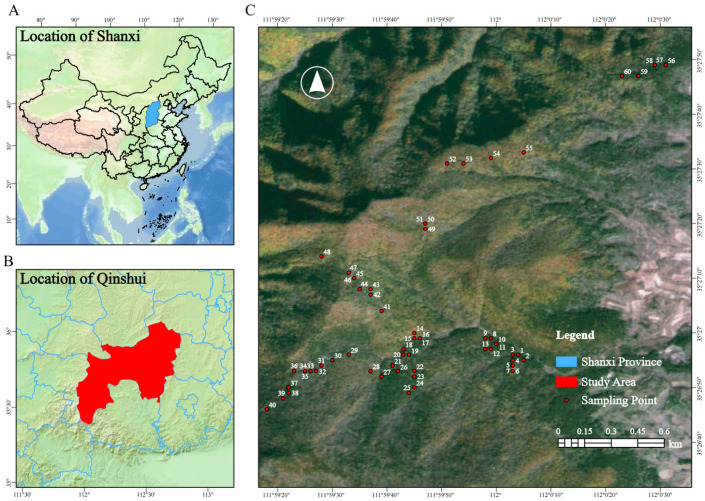
Location of the study area. (**A**) is the location of Shanxi Province, (**B**) is the location of Qinshui County, (**C**) is the location of the sample sites in the study area.

The samples of *D. nipponica* were obtained from Zhongcun Forestry, Zhongtiao Mountain, Shanxi Province (longitude 111°56′ E to 112°14′ E and latitude 35°24′ N to 35°40′ N). It is in a warm temperate semi-humid continental monsoon climate zone. The forest area is 43,810 hm^2^, with an altitude of 1200–1700 m. The average annual temperature is 10.3 °C, and the annual sunshine is 2679.8 h. The frost-free period is about 197 days, and the precipitation is mainly in June to September, with an annual average of 600–800 mm [55,56]. With the support of local research institutions, 60 plots of 10 m × 10 m were randomly set up with the distribution of *D. nipponica* as the center (Figure 5). GPS positioning was carried out for each plot, and the elevation, slope, slope direction, slope position and canopy density of each plot were recorded (Table S1). Five 2 m × 2 m shrub quadrats and 1 m × 1 m herb quadrats were set at the four corners and the center of each plot, respectively. The community information such as species name, diameter at breast height (DBH), ground diameter, height and crown were recorded.

Five sampling points were set up in each plot by diagonal layout, and soil samples of 0–20 cm were collected. The samples in each plot were mixed to obtain representative soil samples. The soil samples were crushed and screened with 18-mesh, 20-mesh and 100-mesh sieves after naturally drying and removing plant debris. In each plot, three rhizome samples were randomly dug. Surface contaminants on the rhizomes samples were removed by washing with deionized water. Rhizomes samples were then oven-dried to constant weight at 85 °C and ground to a powder with stainless steel electric grinder followed by sifting through 100-mesh sieves. The soil samples and rhizome samples were stored in labeled plastic bags at 4 °C pending extraction.

### 4.2. Drought Stress Experimental Design

According to field survey results, the drought stress gradient experiment was carried out in the simulated wild cultivation environment at the Forestry Station of Shanxi Agricultural University. It has a warm temperate continental climate, with an average altitude of 870 m, and the average annual temperature is 10.1 °C. The average annual precipitation is about 410 mm, and the rainfall is mostly concentrated from July to September [7]. The seedling substrate of *D. nipponica* seedlings was plant ash: vermiculite: perlite = 1:1:1. After 6 months, the seedlings of *D. nipponica* with the same growth were selected and planted in the Forestry Station of Shanxi Agricultural University. The diameter of the planting hole was about 15–20 cm, and the depth was about 5 cm. After the soil was covered, we poured the water and carried out the conventional water and fertilizer management.

For the drought stress gradient experiment, the dehydration rate was reduced by 7–10% per day according to the soil water content, and it was reduced to severe drought after 10–12 days. Four water treatments were set up in the experiment, which were control (CK), mild drought (MID), moderate drought (MD) and severe drought (SD) (Table 4). In the event of heavy rainfall, the watering was adjusted by measuring the soil water content after the weather turned clear for 3–5 days. Three replicates were set up in the experiment, with 10 seedlings in each replicate, a total of 120 *D. nipponica* seedlings. Other managements were consistent during the test.

One hundred and twenty days after planting, the survival of seedlings and the proliferation of rhizomes were observed and recorded, and the survival rate of seedlings and the proliferation rate of rhizomes were calculated. The functional leaves of *D. nipponica* were selected, marked and put into self-sealing bags, which were quickly placed in an ice box and brought back to the laboratory to determine their physiological and biochemical indexes, while the photosynthetic physiological parameters were measured on site. For consistency and reliability of the experiment, three healthy seedlings with consistent growth were selected from the three replicate groups of each treatment as technical replicates.

### 4.3. Measurements

#### 4.3.1. Environmental Factors

Environmental factors are mainly divided into biological factors and non-biological factors. In this study, the climatic conditions between different plots are similar, so the investigation of abiotic factors is carried out with geographical differences and soil nutrient differences.

The determination method of soil nutrients was as follows: soil total nitrogen (STN) was determined by Kjeldahl nitrogen determination, soil ammonium hydroxide nitrogen (SAHN) was determined by the alkali solution diffusion method [57], soil organic carbon (SOC) was determined by the K_2_Cr_2_O_7_ oxidative wet digestion method [58], soil available phosphorus (SAP) was determined by sodium bicarbonate extraction method [59].

The calculation method of biological factors is as follows: the dominant species of the community are divided according to abundance [60] and the Shannon–Wiener diversity index [61] and stand density were calculated [62].

#### 4.3.2. Physiological Factors

1. Measurement of gas exchange and pigment content

The determination method of photosynthetic physiological indexes is as follows: the photosynthetic physiological indexes were measured by LI-6400 photosynthetic apparatus at 10:00–15:00, including net photosynthetic rate (Pn), transpiration rate (Tr), intercellular carbon dioxide concentration (Ci), stomatal conductance (Gs) and other indicators. The calculation formula of water use efficiency (WUE) is WUE = Pn/Tr. The environmental factors were atmospheric CO_2_ concentration of (375 ± 20) μmol·mol^−1^, atmospheric temperature of 25–30 °C and relative humidity of (35 ± 8)%.

About 200 mg fresh leaves were placed in a mortar with a small amount of quartz sand and 95% ethanol after crushing to form a homogenate. Then it was filtered into a 25 mL volumetric flask and diluted with ethanol to volume. Using a spectrophotometer, the absorbance of the pigment extracts was measured at 646 and 663 nm to assess total chlorophyll content [63].

2. Measurement of antioxidant enzyme activities

About 500 mg fresh leaves were placed in a frozen mortar with phosphate buffer (pH 7.8, 50 mmol·L^−1^) that had been prechilled. Then the homogenate was then centrifuged at 4000 r·min^−1^ for 10 min at 4 °C. The filtrate was obtained as an enzyme extract for the determination of superoxide dismutase (SOD) and catalase (CAT) activities. SOD activity was determined by the NBT method [64]. CAT activity was determined by potassium permanganate titration [65].

3. Measurement of malondialdehyde contents

About 500 mg fresh leaves were placed in a mortar with 10 mL of 10% trichloroacetic acid (TCA) to form a homogenate. The homogenate was then centrifuged at 4000 r·min^−1^ for 10 min at 4 °C, then 2 mL of 0.6% thiobabutrate (TBA) was added to the supernatant. The mixture was left to stand at 100 °C for 15 min before centrifugation again, and the absorbance values of the supernatant were recorded at 532 nm, 600 nm and 450 nm [66].

4. Measurement of soluble protein, soluble sugar and proline contents

Soluble protein (SP) content was determined by the G-250 Coomassie brilliant blue method [27]. About 500 mg fresh leaves were placed in a mortar with 5 mL phosphate buffer (pH 7.8, 50 mmo·L^−1^) to form a homogenate. The mixture was centrifuged at 4000 r·min^−1^ for 10 min at 4 °C, then 0.1 mL supernatant was mixed with Coomassie brilliant blue G-250 solution. After standing for 2 min, the absorbance values of the supernatant were recorded at 595 nm.

Soluble sugar (SS) content was determined by the anthrone ethyl acetate colorimetric method [27]. About 500 mg of dried leaf powder was mixed with 10 mL of 75% ethanol in a 95 °C water bath for 1 h. After the mixture was cooled and filtered, the supernatant was diluted to 50 mL with 75% ethanol to volume, 1 mL of the supernatant was added with 4 mL of anthrone ethyl acetate reagent (20 g·L^−1^) and the mixture was heated in a 100 °C water bath for 8 min. The absorbance of the solution at 630 nm was recorded after cooling.

Proline (Pro) content was determined by the ninhydrin method [27]. About 500 mg fresh leaves were placed with 5 mL 3% sulfosalicylic acid mixed at 100 °C in a water bath for 15 min and centrifuged at 4000 r·min^−1^ for 10 min after cooling. Then, 1 mL of supernatant was added to 1 mL of glacial acetic acid, 1 mL of water and 2 mL of acidic ninhydrin reagent in a 100 °C water bath for 40 min. After cooling, 5 mL of toluene was added, shaken and left to stand in the dark for 20 min. The absorbance of the toluene layer at a 520 nm wavelength was recorded.

#### 4.3.3. Diosgenin

About 200 mg dried rhizome powder was placed in a 60 mL round-bottom flask. Then 10 mL of 1.5 mol/L hydrochloric acid–methanol hydrolysate and 10 mL of petroleum ether were added and heated in a water bath at 85 °C for reflux extraction for 3–4 h. After the reaction, the solution was cooled, transferred to a 50 mL centrifuge tube, added with 10 mL of petroleum ether and centrifuged at 2000 r·min^−1^ for 5 min. The supernatant was placed in a new centrifuge tube. The extract was dried, redissolved in 2 mL methanol solution (HPLC), filtered through a 0.22 μm needle-type microporous filter and the filtrate was collected. The preparation of the test solution was completed.

Detection was performed using an EClassical 3200 High Performance Liquid Chromatograph (Dalian Elite Analytical Instruments Co., Ltd., DLC, Dalian, China) (C18 reverse, 250 mm × 4.6 mm, 10 μL). The conditions for detecting the content of diosgenin by HPLC were as follows: the mobile phase was acetonitrile and 0.04% acetic acid aqueous solution (90:10), the flow rate was 1 mL·min^−1^, the column temperature was 30 °C and the detection wavelength was 203 nm.

### 4.4. Statistical Analysis

Statistical analyses were conducted in R (v4.4.3). The effects of environmental factors on Number, Height, Diameter, DW and Moisture were quantified by multiple regression models [67,68]. The above growth indexes were analyzed by detrended correspondence analysis (DCA), and the gradient axis length (LGA) was calculated to test whether the analysis model was linear or non-linear. Because LGA < 3, the redundancy analysis (RDA) based on the linear model was used to analyze the influence of environmental factors on the growth indexes of *D. nipponica*. The model avoided over-fitting caused by collinearity through ridge regression regularization. *R*^2^ was used to test the goodness of fit of the model, and the significance of marginal effects and conditional effects was determined by a Monte Carlo permutation test [69].

The XGBoost-SHAP model was used to explore the effects of growth indicators and environmental factors on the content of diosgenin. Before the model fitting, each index was numerically normalized (Table S2). Extreme gradient boosting (XGBoost) is an advanced ensemble learning method based on the gradient boosting framework of the decision tree algorithm [70]. The decision tree algorithm is less sensitive to data. Compared with GLM, the decision tree model is more suitable for analyzing complex ecological data [71] and is superior to many traditional machine learning methods in dealing with complex features and non-linear relationships [72]. The XGBoost-SHAP framework combines the predictive power of XGBoost and the interpretability of SHAP. SHAP based on game theory assigns a value to each feature to quantify its contribution to the prediction, indicating the positive or negative impact of the feature on the model output [73]. The configuration of the model training parameters is as follows: the number of learners is set to 44, and the number of iterations is 50, 100, 200 and 300. The maximum tree depth is set to 2–11, and the learning rate is set to 0.1, 0.3 and 0.5. Gamma is set to 0, 0.25 and 0.5. The learning task type is set to xgbTree, and the random seed is fixed to 123 [74]. The model avoids over-fitting through cross-validation. *R*^2^, MSE, RMSE, MAE and MAPE were used to test the goodness of fit of the model [75].

The one-way ANOVA combined with the least significant difference (LSD) multiple comparison method was used to analyze the differences in physiological parameters of *D. nipponica* under different treatments. Finally, the results of PLS-PM are verified and supplemented to illustrate the relationship between factors in field and laboratory experiments. PLS-PM tests the direction and intensity of causality between potential variables in the form of path coefficients [76]. This approach analyzed potential direct and indirect effects among these factors and content of diosgenin in rhizomes of *D. nipponica*. In PLS-PM, the direct effects are represented by the corresponding path coefficients, the indirect effects are the paths containing intermediate variables and the total effect is the sum of the direct and indirect effects describing the relationship between the variables. The fitness of PLS-PM is indicated by the coefficient of determination (*R*^2^) and the goodness of fit (GoF) [77].

## 5. Conclusions

In this study, the XGBoost-SHAP model was used to fit the non-linear relationship between multi-type factors and the content of diosgenin in the rhizome of wild *D. nipponica*. The main factors affecting the content of diosgenin were found from 21 factors in different aspects such as geographical factors, community factors, soil nutrients and growth indicators. The results showed that water-related factors (Moisture and Slope) had the greatest effect on the total synthesis of diosgenin. In addition, STN and SAHN were also closely related to the content of diosgenin. The RDA model showed that SAP and STN were the main environmental factors affecting the growth of *D. nipponica*. Faced with different degrees of drought stress, the growth and metabolism of *D. nipponica* were significantly affected. Mild stress (MID) promoted the biomass accumulation and diosgenin synthesis of *D. nipponica*, while severe stress (SD) inhibited the growth and metabolism of *D. nipponica* but promoted its proliferation. The results of this study provide a useful basis for improving the imitation wild cultivation and breeding period monitoring of *D. nipponica*. However, if we want to clarify the internal mechanism of mild drought to increase the content of diosgenin, we still need to carry out independent control experiments to verify, which will be the core of our next work.

## Figures and Tables

**Table 1:** One-way ANOVA for different drought stress levels on the growth indexes of *D. nipponica*.

Drought Treatment	SR	PR	Height (cm)	Diameter (cm)
CK	0.81 ± 0.03 b	0.24 ± 0.02 b	10.53 ± 1.07 a	0.61 ± 0.11 ab
MID	0.92 ± 0.07 a	0.18 ± 0.03 c	12.30 ± 1.14 a	0.78 ± 0.10 a
MD	0.65 ± 0.05 c	0.28 ± 0.01 b	11.27 ± 1.41 a	0.52 ± 0.10 b
SD	0.54 ± 0.02 d	0.33 ± 0.02 a	11.06 ± 1.03 a	0.51 ± 0.12 b
*p*-value	*p* < 0.001 ***	*p* < 0.001 ***	0.3688	0.05745

Values are mean ± standard deviation (*n* = 3). Values within the same columns followed by different letters are significantly different (*p* < 0.05). Asterisks denote significance levels (*** *p* < 0.001). SR, seedling survival rate; PR, rhizome proliferation rate; Height, plant height; Diameter, ground diameter.

**Table 2:** One-way ANOVA for different drought stress levels on the physiological parameters of *D. nipponica*.

Drought Treatment	CK	MID	MD	HD	*p*-Value
SOD (U·g^−1^FW)	85.98 ± 3.17 d	178.20 ± 13.97 a	152.37 ± 2.34 b	114.48 ± 1.59 c	*p* < 0.001 ***
CAT (U·g^−1^FW)	1.33 ± 0.01 d	1.44 ± 0.03 c	1.67 ± 0.01 b	1.98 ± 0.01 a	*p* < 0.001 ***
MDA (μmol·g^−1^FW)	4.35 ± 0.23 c	5.18 ± 0.80 bc	7.61 ± 2.57 b	12.27 ± 0.71 a	*p* < 0.001 ***
Pro (μg·mL^−1^FW)	53.44 ± 4.89 d	76.37 ± 0.21 c	95.58 ± 2.56 b	118.51 ± 3.08 a	*p* < 0.001 ***
SS (%DW)	0.29 ± 0.02 c	0.45 ± 0.01 a	0.45 ± 0.04 a	0.36 ± 0.01 b	*p* < 0.001 ***
SP (mg·g^−1^FW)	7.90 ± 0.18 b	8.49 ± 0.12 a	7.60 ± 0.07 b	6.50 ± 0.41 c	*p* < 0.001 ***
Chl (mg·g^−1^FW)	3.67. ± 0.28 c	4.46 ± 0.01 b	4.77 ± 0.01 a	4.65 ± 0.01 ab	*p* < 0.001 ***
Pn (μmol·m^−2^·s^−1^)	4.28 ± 0.59 a	3.18 ± 0.41 b	1.76 ± 0.30 c	0.70 ± 0.30 c	*p* < 0.001 ***
Tr (mmol·m^−2^·s^−1^)	0.77 ± 0.1 a	0.64 ± 0.03 a	0.43 ± 0.05 b	0.28 ± 0.11 b	*p* < 0.001 ***
Gs (μm)	3.74 ± 0.55 a	2.53 ± 0.43 b	1.69 ± 0.37 bc	1.03 ± 0.44 c	*p* < 0.001 ***
Ci (μmol·mol^−1^)	99.33 ± 2.08 d	154.67 ± 5.03 c	218.67 ± 12.10 b	295.00 ± 13.75 a	*p* < 0.001 ***
WUE (μmol·mmol^−1^)	3.74 ± 0.46 ab	4.49 ± 0.75 a	3.07 ± 0.54 bc	2.48 ± 0.28 c	*p* < 0.01 **

Values are mean ± standard deviation (*n* = 3). Values within the same rows followed by different letters are significantly different (*p* < 0.05). Asterisks denote significance levels (** *p* < 0.01, *** *p* < 0.001). SOD, superoxide dismutase; CAT, catalase; MDA, malonaldehyde; Pro, proline; SS, soluble sugar; SP, soluble protein; Chl, chlorophyll; Pn, net photosynthetic rate; Tr, transpiration rate; Gs, stomatal conductivity; Ci, CO_2_ concentration between cells; WUE, water use efficiency.

**Table 3:** One-way ANOVA for different drought stress levels on the growth indexes of rhizomes of *D. nipponica*.

Drought Treatment	DW (g)	Moisture (%)	Dio (mg·g^−1^)
CK	0.58 ± 0.16 c	8.02 ± 0.35 b	3.2 ± 0.3 b
MID	0.79 ± 0.15 a	8.50 ± 0.34 a	6.5 ± 0.1 a
MD	0.66 ± 0.14 b	7.91 ± 0.56 c	2.3 ± 0.3 c
SD	0.62 ± 0.14 bc	7.68 ± 0.58 d	1.1 ± 0.2 d
*p*-value	*p* < 0.001 ***	*p* < 0.001 ***	*p* < 0.001 ***

Values are mean ± standard deviation (*n* = 3). Values within the same columns followed by different letters are significantly different (*p* < 0.05). Asterisks denote significance levels (*** *p* < 0.001). DW, rhizome dry weight; Moisture, water content of rhizome; Dio, content of diosgenin in rhizome.

**Table 4:** The process of drought stress treatment.

Drought Treatment	CK	MID	MD	SD
The interval of watering/d	3	5	8	10
The relative soil water content/%	70–80	50–60	35–50	20–35
Drought degree	No drought	Mild drought	Moderate drought	Severe drought

## Data Availability

Data will be made available on request.

## Supplementary Materials

The following supporting information can be downloaded at: https://www.mdpi.com/article/10.3390/plants14192998/s1, Table S1: Basic situation of *D. nipponica* sample plot; Table S2: Multi-type environmental factors and *D. nipponica* data pretreatment.

## Abbreviations

The following abbreviations are used in this manuscript:

Position	Slope position
STN	Soil total nitrogen
SAHN	Soil ammonium hydroxide nitrogen
SAP	Soil available phosphorus
SOC	Soil organic carbon
C/N	Carbon to nitrogen ratio
N/P	Nitrogen to phosphorus ratio
C/P	Carbon to phosphorus ratio
Type	Community type
SD	Stand density
CC	Canopy closure
SC	Shrub cover
HC	Herbaceous cover
Diversity	Community diversity
Count	Community stem count
Height	Plant height
Diameter	Ground diameter
DW	Dry weight of rhizome
Moisture	Moisture content of rhizome
Dio	Diosgenin content of rhizome
SR	Seedling survival rate
PR	Rhizome proliferation rate
SOD	Superoxide dismutase
CAT	Catalase
MDA	Malonaldehyde
Pro	Proline
SS	Soluble sugar
SP	Soluble protein
Chl	Chlorophyll
Pn	Net photosynthetic rate
Tr	Transpiration rate
Gs	Stomatal conductivity
Ci	CO_2_ concentration between cells
WUE	Water use efficiency
