# Supplementary material for: Mild Drought Promotes Biomass Accumulation and Increases Diosgenin Content in Rhizomes of Dioscorea nipponica
Source: Plants (Basel). 2025 Sep 28;14(19):2998. doi: 10.3390/plants14192998 (PMC12526088; doi:10.3390/plants14192998)
Supplement: Supplementary file 1 [file plants-14-02998-s001.zip › plants-3879913-supplementary.pdf]

Light drought promotes biomass accumulation and increases diosgenin content in rhizomes of *Dioscorea nipponica*

Supplementary material

Table S1 Basic situation of *D.nipponica* sample plot.

Plot no.	Longitude	Latitude	Elevation/m	Slope/°	Slope position	Aspect	Canopy density
1	112°0′4″	35°26′56″	1635	20	45	Downhill	0.67
2	112°0′5″	35°26′55″	1640	29	47	Level slope	0.6
3	112°0′3″	35°26′56″	1639	25	26	Level slope	0.65
4	112°0′3″	35°26′55″	1640	26	19	Moderately uphill	0.88
5	112°0′3″	35°26′54″	1650	11	28	Uphill	0.56
6	112°0′3″	35°26′53″	1670	26	15	Uphill	0.77
7	112°0′3″	35°26′53″	1670	12	39	Uphill	0.82
8	111°59′59″	35°26′59″	1650	31	44	Downhill	0.7
9	111°59′58″	35°26′59″	1650	34	44	Level slope	0.85
10	111°59′60″	35°26′58″	1650	25	55	Downhill	0.85
11	111°59′60″	35°26′58″	1650	30	47	Downhill	0.73
12	111°59′59″	35°26′57″	1660	10	127	Level slope	0.69
13	111°59′58″	35°26′57″	1670	12	70	Level slope	0.55
14	111°59′45″	35°26′60″	1660	40	163	Downhill	0.73
15	111°59′45″	35°26′59″	1670	49	150	Downhill	0.88
16	111°59′46″	35°26′59″	1670	49	194	Downhill	0.57
17	111°59′46″	35°26′59″	1670	8	181	Uphill	0.59
18	111°59′43″	35°26′57″	1680	12	150	Uphill	0.55
19	111°59′44″	35°26′56″	1680	19	330	Level slope	0.86
20	111°59′43″	35°26′56″	1680	17	150	Uphill	0.66
21	111°59′41″	35°26′54″	1671	9	6	Downhill	0.6
22	111°59′45″	35°26′53″	1672	11	29	Moderately uphill	0.75
23	111°59′45″	35°26′52″	1682	20	43	Moderately uphill	0.75
24	111°59′45″	35°26′50″	1690	12	31	Moderately uphill	0.55
25	111°59′44″	35°26′49″	1700	14	52	Moderately uphill	0.6
26	111°59′42″	35°26′53″	1700	36	2	Uphill	0.71
27	111°59′39″	35°26′52″	1676	25	352	Uphill	0.79
28	111°59′37″	35°26′53″	1687	60	67	Uphill	0.8
29	111°59′33″	35°26′56″	1700	26	23	Uphill	0.87
30	111°59′30″	35°26′55″	1709	34	351	Uphill	0.83
31	111°59′28″	35°26′54″	1726	35	77	Uphill	0.59
32	111°59′27″	35°26′53″	1720	13	45	Downhill	0.6
33	111°59′26″	35°26′53″	1745	20	66	Downhill	0.5
34	111°59′25″	35°26′53″	1747	21	89	Uphill	0.8

35	111°59′25″	35°26′53″	1780	21	352	Downhill	0.83
36	111°59′23″	35°26′53″	1759	19	117	Uphill	0.84
37	111°59′22″	35°26′50″	1800	22	316	Downhill	0.66
38	111°59′22″	35°26′49″	1820	24	312	Downhill	0.61
39	111°59′21″	35°26′48″	1810	33	325	Downhill	0.7
40	111°59′18″	35°26′46″	1820	41	322	Downhill	0.7
41	111°59′39″	35°27′4″	1749	40	142	Uphill	0.74
42	111°59′37″	35°27′7″	1736	42	72	Uphill	0.74
43	111°59′37″	35°27′8″	1730	42	70	Uphill	0.55
44	111°59′35″	35°27′8″	1747	32	55	Downhill	0.61
45	111°59′34″	35°27′10″	1750	30	79	Downhill	0.8
46	111°59′34″	35°27′10″	1731	31	70	Uphill	0.61
47	111°59′33″	35°27′11″	1750	33	70	Uphill	0.64
48	111°59′28″	35°27′14″	1737	38	197	Uphill	0.59
49	111°59′47″	35°27′19″	1700	32	137	Uphill	0.8
50	111°59′47″	35°27′20″	1714	16	137	Uphill	0.77
51	111°59′47″	35°27′20″	1711	30	120	Uphill	0.82
52	111°59′51″	35°27′31″	1640	43	193	Uphill	0.62
53	111°59′54″	35°27′31″	1652	26	170	Uphill	0.56
54	111°59′59″	35°27′32″	1670	38	168	Uphill	0.73
55	112°0′5″	35°27′33″	1680	35	180	Uphill	0.67
56	112°0′31″	35°27′49″	1600	26	40	Downhill	0.86
57	112°0′29″	35°27′49″	1616	17	8	Downhill	0.4
58	112°0′29″	35°27′49″	1611	30	358	Downhill	0.56
59	112°0′26″	35°27′47″	1734	46	347	Downhill	0.58
60	112°0′23″	35°27′47″	1596	27	40	Downhill	0.64

Table S2 Multi-type environmental factors and *D.nipponica* data pretreatment.

Factor types	Index	Abbreviation	Original value	Processed values
Geography	Altitude	Altitude	1596-1820 m	Range from 0 to 1 after normalization
	Slope	Slope	8-60 °	Range from 0 to 1 after normalization
	Aspect	Aspect	Range from 2 to 358	Range from 0 to 1 after normalization
	Slope position	Position	Uphill; Moderately uphill; Level slope; Moderately downhill; Downhill	Assigned at 1,2,3,4,5 after Encoding
Soil properties	Soil total Nitrogen	STN	1.64-4.93 $g \cdot kg^{-1}$	Range from 0 to 1 after normalization
	Soil Ammonium Hydroxide Nitrogen	SAHN	13.2-40.53 $mg \cdot kg^{-1}$	Range from 0 to 1 after normalization
	Soil Available Phosphorus	SAP	0.55-9.98 $g \cdot kg^{-1}$	Range from 0 to 1 after normalization
	Soil Organic Carbon	SOC	18.05-66.38 $g \cdot kg^{-1}$	Range from 0 to 1 after normalization
	Carbon to Nitrogen Ratio	C/N	Range from 6.52 to 27.99	Range from 0 to 1 after normalization
	Nitrogen to Phosphorus Ratio	N/P	Range from 0.26 to 3.84	Range from 0 to 1 after normalization
	Carbon to Phosphorus Ratio	C/P	Range from 2.49 to 36.43	Range from 0 to 1 after normalization
Community ecology	Community type	Type	Broad leaf forest; Mongolian oak forest; Conifer forest; Pine-oak mixed forest; *Forsythia-Spiraea* shrub; *Forsythia* shrub	Assigned at 1,2,3,4,5,6 after Encoding
	Stand density	SD	Range from 0 to 29	Range from 0 to 1 after normalization
	Canopy closure	CC	Range from 0.40 to 0.88	Range from 0 to 1 after normalization
	Shrub cover	SC	Range from 0.84 to 47.80	Range from 0 to 1 after normalization
	Herbaceous cover	HC	Range from 0.42 to 8.41	Range from 0 to 1 after normalization
	Community diversity	Diversity	Range from 0.69 to 0.93	Range from 0 to 1 after normalization
Growth condition	Community stem count	Count	1-24	Range from 0 to 1 after normalization
	Plant Height	Height	29.25-283.33 cm	Range from 0 to 1 after normalization
	Ground Diameter	Diameter	0.13-0.40 cm	Range from 0 to 1 after normalization
	Dry Wight	DW	1.18-66.13 g	Range from 0 to 1 after normalization
	Moisture Content	Moisture	0.74-47.19%	Range from 0 to 1 after normalization
	Diosgenin	Dio	0.13-1.29 $mg \cdot g^{-1}$	Primitive value
